# Hue Contrast and the Sense of Space

**DOI:** 10.1068/i0701

**Published:** 2015-04-01

**Authors:** Jan Koenderink, Andrea van Doorn, Liliana Albertazzi, Johan Wagemans

**Affiliations:** Laboratory of Experimental Psychology, University of Leuven (KU Leuven), Leuven, Belgium; and Faculteit Sociale Wetenschappen, Psychologische Functieleer, Universiteit Utrecht, Utrecht, The Netherlands; Faculteit Sociale Wetenschappen, Psychologische Functieleer, Universiteit Utrecht, Utrecht, The Netherlands; CIMeC, Palazzo Fedrigotti, Rovereto, Italy; and Department of Humanities, University of Trento, Trento, Italy; Laboratory of Experimental Psychology, University of Leuven (KU Leuven), Leuven, Belgium

**Keywords:** brightness, depth perception, hue, individual differences, landscapes, microgenesis, templates

## Abstract

Does human vision deploy a generic template for open landscapes that might fit the gist of current optical input? In an experiment, participants judged depth order in split-field images in which the two fuzzily delineated half-images were filled with different hues. For the majority of observers, we find a systematic dependence of depth order of these half-images on their hue and/or brightness difference. After minor cleaning of the data, we are left with two mutually well-separated clusters. Correlation with the statistical distribution of hue and brightness in generic “open landscape” photographs reveals that one cluster correlates with hue, the other with brightness. This suggests that human observers indeed at least partly rely on “generic landscape” templates in the psychogenesis of their visual awareness.

## 1 Introduction

**Figure 1.**
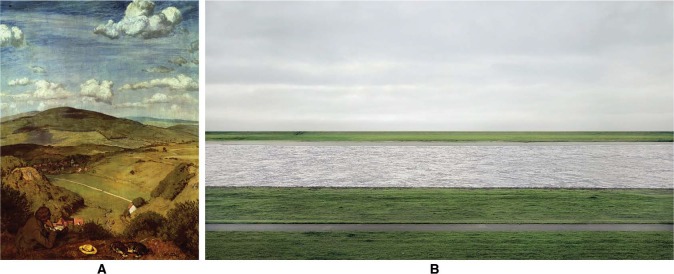
At left a painting by Hans Thoma (1839–1924; “Landscape at Taunus.” 1890). At right a photograph by Andreas Gursky (born 1955).

In landscape renderings (examples in Figure 1). one often spontaneously notices two generic properties—apart from the obvious fact that both depict landscapes:

- there is a spatial organization in terms of horizontal bands,
- these bands are varicolored in a certain order of hues.

The austere banding was created by Photoshopping in the case of Andreas Gursky's photograph (Figure 1 right). In Hans Thoma's painting (Figure 1 left). the banding is also a dominant aspect of the composition. This painting also illustrates the canonical color scheme that one encounters in Western landscape painting, namely roughly a brown-foreground, a green-middle ground, and a blue-background (Clark. 1949; Gurney. 2010). Moreover, one usually encounters a brightness gradient approximately running from dark (below) to light (top). The omnipresence of this scheme in the arts suggests that it may be a template in the psychogenesis of visual awareness.

**Figure 2.**
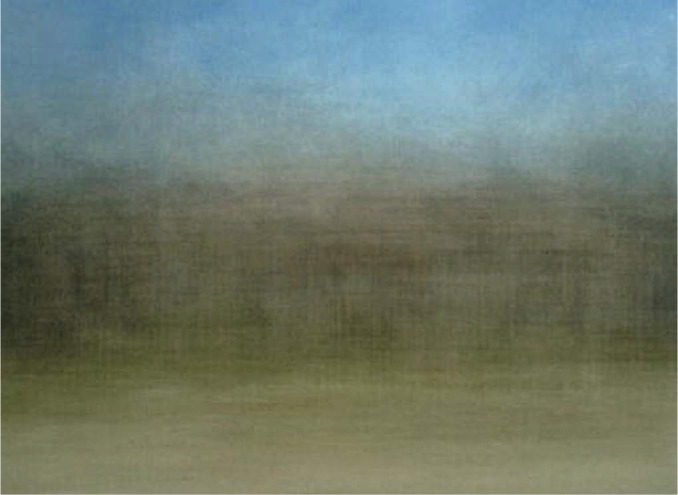
Jason Salavon “Homes for sale” (2002) shows the average of 112 realtor photos of single-family homes for sale in Miami-Dade county.

Indeed, abstract paintings that show the banding and the colors readily call landscapes to mind. This is exploited by the artist Jason Salavon, who extracts the colors and banding for a common core of about a hundred photographs (see Figure 2). The detailed content of the individual images is lost, the average merely contains “the gist” of such pictures (Oliva & Torralba. 2006). the gist being an abstract entity that might well be called a “generic landscape.”

**Figure 3.**
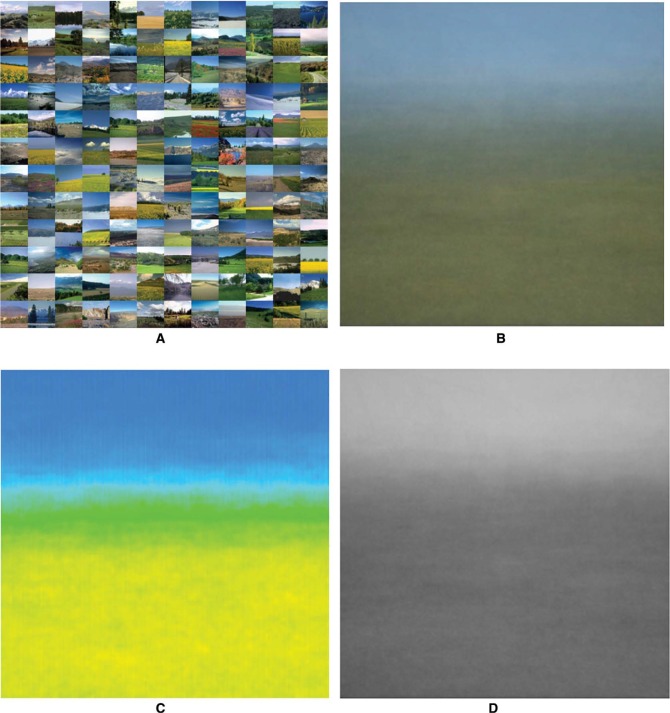
At top left 144 “open country” images from Torralba's SUN database (Xiao et al, 2010). At top right the average image, at bottom left the corresponding full colors (see Appendix B), at bottom right the corresponding brightness. Both the banding and the color scheme are immediately apparent. The average landscape is blue and light on top, yellow and dark at bottom. Notice that reds and purples do not appear in the overall average.

Antonio Torralba and Aude Oliva (Xiao. Hays. Ehinger. Oliva. & Torralba. 2010) consider such average, gist-like structures in the context of automatic image interpretation. We thankfully exploited their database of 256 × 256 pixel images randomly collected from the Internet, selecting the ones labeled “open country.” Averaging even small subsets immediately reveals the banding and the color scheme. For clarity, we also show the average as “full colors” (Appendix B). which are the colors without their white and black content (see Figure 3).

**Figure 4.**
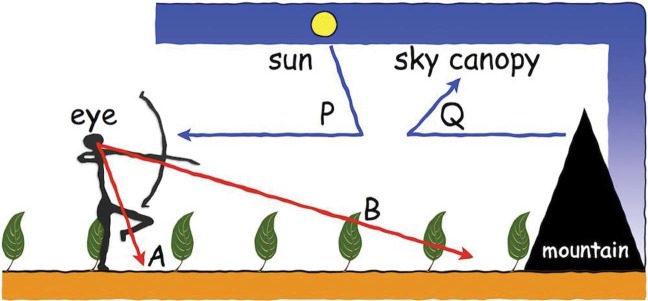
The human optical condition in a Savannah landscape. Notice that visual ray A hits the soil, whereas visual ray B is intercepted by the vegetation. The light ray from the distant mountain Q never makes it to the eye because it is scattered out of the beam. (Notice that “light” and “visual” rays have opposite directions!) In contradistinction, the light ray from the sun P is scattered into the beam and reaches the eye from the direction of the mountain. The sky canopy fills the upper part of the visual field. This simple scheme captures most of the relevant optics of an early human Umwelt (von Uexküll, 1921).

The physical reasons for the banding are obvious enough (see Figure 4). In a generic open landscape, the scene is divided along the horizon into ground and sky. The sky canopy tends to be blueish, the “sky blue” being due to Rayleigh scattering (see below). Such an environment has probably been important in human evolution (Cerling et al., 2011; Stringer. 2011). The ground is low in a typical image, whereas the sky is high in the image. A part of the ground is seen at a distance that is roughly inversely proportional to its horizon dip, and viewed at an oblique angle that is proportional to its horizon dip. Near your feet, you see earth, at a distance you see vegetation. That is why “the grass is greener at the other side of the fence”; this is a pure perspective effect, already described by Leonardo for the case of colonnades (Pirenne, 1970).

An additional factor in the natural color scheme is the “air light” (Koschmieder, 1924). The discrete structure of air—mainly a mixture of 78% N_2_ (nitrogen) and 21% O_2_ (oxygen) molecules—implies that there are appreciable density fluctuations in volumes of about a cubed wavelength of electromagnetic radiation in the visual band. This implies refractive index fluctuations, and thus a scattering of the radiation. The scattering cross section is proportional to the inverse fourth power of the wavelength (Strutt, that is Lord Rayleigh, 1871a, 1871b, 1871c, 1899). The effects of the air light are that the color of dark objects, that is the generic case, approaches the color of the horizon sky. In clear air that means blueish. In contradistinction, the color of (very) light objects approaches its complement, in clear air yellow to red. Thus, distant mountains will appear blue, whereas the setting sun will appear red (Middleton, 1952; Minnaert, 1954).

Because of pure perspective effects alone, one predicts the horizontal banding. Since the earth colors are dark reds and yellows, the vegetation colors green, yellows, and occasional reds, and the maximum of the Rayleigh scattering cross section lies in the blue, one predicts a color scheme from yellow-brown, over green-yellow, to whitish-blue, and finally deep blue, as one goes from bottom to top in the visual field.

Some colors, like reds and purples, do not figure at all in this overall scheme. They can easily be fitted in though. Because they mainly occur as the colors of smallish details, such as flowers, they can only be expected in the lower parts of the image. Moreover, due to the air light, purples are bound to occur above reds in the visual field. One expects their abundance to be very variable, unlike the abundances of the yellows and blues, which are largely fixed.

Whereas the ecological physics is thus fairly trivial, the qualities and meanings in visual awareness are hardly understood, but certainly interesting. It seems likely that the psychogenesis of visual awareness involves a template-like gist that “means” something like a generic open country, perhaps of the savanna variety. Since this is supposedly where early man evolved, this is perhaps not too surprising from a biological perspective (Dutton, 2003; Hilbert, 1992; Hilbert & Byrne, 2011; Matthen, 1988; Mollon, 1989).

From the viewpoint of theoretical ethology one might consider visual awareness to be an “optical user interface” that maximizes our biological fitness (Hoffman, 2009). Thus, visual awareness is not about “representing the physical world.” Rather the opposite: *effective interfaces shield the user from unnecessary complexity.* The interface is made up of abstract objects (Meinong, 1899) and internal functional relations, that are constructions of the mind, conducive to efficacious action planning. This is the “counter world” as proposed by Jakob von Uexküll (1921). The “open landscape” object may well be a “generic gist.” or template, that is of use to start off the psychogenesis. Such a notion is consistent with a number of recent theories in neuroscience that propose that high-level templates are generated quickly from a fast-feedforward sweep (e.g., Bar, 2003; Bar et al., 2006; Cerling et al., 2011; Hegdé, 2008; Hochstein & Ahissar, 2002).

According to the neurologist Brown (2002). the psychogenetic process is best understood to originate from hallucinations, or dreamlike states that progressively diversify and mutually compete in an evolutionary process, so as to eventually “account for” the neural activity in the visual areas, with the primary visual cortex as “end station.” This is “controlled hallucination.” or “analysis by synthesis” according to whether you are from a psychological or engineering background (Neisser, 1976; for some computational models of psychogenesis, see Beal & Sussman, 2008; Bever & Poeppel, 2010; Yuille & Kersten, 2006). The final “visual objects” in visual awareness are like the rigidified crust of this process, unfit to further articulation. They are experienced as objects of reality. Perhaps perversely, mainstream thought considers them as the *causes* of visual awareness, putting reality on its head, so to speak.

All this may serve to explain the focus of the present research. We are interested in whether visual psychogenesis indeed deploys landscape-like, gist-like template structures. We intend to investigate this through abstract, at first blush unlandscape-like test images. This avoids possible interference with semantic associations, and specific memories.

## 2 Methods

In this study, we have to depend upon a large group of inexperienced observers. It was desirable to have these naïve observers use binocular viewing, as this saves session time, and is very likely to decrease the fraction of inevitably unreliable results. However, the chromostereopsis effect (Kishto. 1966) might conceivably necessitate the use of monocular viewing. (See below.) Hence, we piloted with three experienced observers of which two are very familiar with the effects of chromostereopsis and do experience its generic effect, whereas the third is experienced in many kinds of visual psychophysics (certainly not “naive”). As expected in our stimulus configuration (see below), we did not find any significant differences between monocular and binocular viewing. Thus, we decided on binocular presentation.

### 2.1 Participants

The participants were 15 students from Trento University who participated in order to gain credits for their study program, as well as 20 postgraduate students, postdocs, and staff from the University of Leuven, who participated as volunteers. In all cases, we obtained affirmed consent. Thus, our study is based on a total of 35 participants.

### 2.2 Experimental procedure

The stimuli were presented on an LCD screen measuring 15″ over the diagonal, subtending 1,280 × 800 pixels. The screen was calibrated and linearized via the SuperCal program (BergDesign, 2014). Details of the calibration can be found in Appendix A.

The screen background pattern had 1,500 polygonal cells of random shape and gray values. The observers sat at 57 cm from the screen in an otherwise darkened room. Viewing was binocular.

The stimuli were 500 pixels square, that is 12.5° square in the visual field, positioned at the center of the screen. The response device was a keyboard.

There were necessarily (very) minor physical differences between the Trento and Leuven setups, but these are unlikely to be of importance. The task is a very global one, and minor details of the procedure are not likely to matter much.

### 2.3 Stimulus configuration

**Figure 5.**
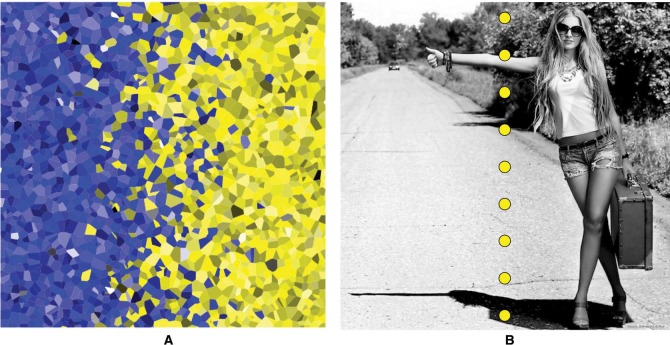
Left: One of the stimulus patterns. In this one, the separation (“fuzzy edge”) is vertical, with the yellow on the right. In the experiment, this pattern will reappear four times, with the yellow on the right side (shown), the top side, the left side, and the bottom side. Correct responses are “left side” and “right side.” Thus, “top side” or “bottom side” indicate “sloppiness” in this example. Right: One of the catch trials. The stippled line divides the two panes, taking over the role of the hue boundary. Here, the correct response is “top side.” Anything else indicates “sloppiness.”

A typical stimulus image is shown in Figure 5 left. It is made from a texture of random, convex polygons, randomly filled with either of two colors, the probabilities changing gradually so as to form a fuzzy, ragged edge. Such a configuration is somewhat reminiscent of a painting, it looks “material,” whereas a two-panel array of flat colors would look flimsy and immaterial in comparison. In Figure 5 left, the fuzzy edge is vertical. Each such pattern will reappear four times, with the yellow on the right (shown), the top, the left, and the bottom.

The basic patterns are defined by a pair of colors. These colors are selected from the set {yellow, yellow-green, green, green-cyan, cyan, cyan-blue, blue, blue-magenta, magenta, magenta-red, red, red-yellow}, all pairs being presented in equal numbers. The RGB-coordinate values are either 0, 0.5, or 1. Notice that red-yellow = {1, 0.5, 0} is usually called orange, and so forth, but we are not interested in color naming issues here. Thus, there are 4 × 12(12-1)/2 = 264 distinct stimulus pairs. The stimuli were presented in an overall randomized order.

For the 20 observers of the Leuven group, the regular trials were interspersed with 10% catch trials (Figure 5 right). The catch trials were monochrome photographs in which the nearest image side was immediately obvious because the division into two “panels” was made graphically explicit (Figure 5 right). There were catch trials for each fuzzy edge, and the catch trials were presented in all four orientations. They were randomly intermixed between the regular trials.

As all observers got all catch trials right, these were not further used in the analysis. Importantly, this indicates that the observers indeed understood the task.

### 2.4 Task

The task of the observer was to decide which side of the square is closest, and to indicate this judgment by way of the corresponding left, right, up, or down arrow keys. Observers were asked to react immediately, on their gut feeling, since their reflective thought would be useless anyway. They were told to proceed at their own pace, and that they would be presented with hundreds of instances—this serves to discourage reflective thought. In practice, they went quickly through all trials, and, in retrospect, and according to their reactions, apparently enjoyed it. The patterns appear visually attractive.

Perhaps surprisingly, no one complained that the task made no sense. After all, it really does not make much sense, for it is far from obvious why one color should appear “closer” than another. When observers come up with essentially random answers that would be fully acceptable to us.

### 2.5 Experimental data

The single-observer data from a session yields 264 answers in the range {right, top, left, bottom}. We attempt to account for the results through a linear order of “closeness” of the 12 colors.

Finding the best linear order for a full set of pairwise comparisons is a standard problem. Of course, such a best order is not likely to account for the data exactly, reason being that one attempts to account for 264 answers through only 11 degrees of freedom. We quantify this through an appropriate figure of merit.

From a simulation we know the distribution of this figure of merit for a randomly responding observer, which enables us to judge the significance of a result. It is an objective measure of the self-consistency of an observer's response. We present further detail below.

## 3 Results

A total of 35 observers completed the experiment. Their data can be checked in various ways. For instance, since it is always visually obvious whether the “fuzzy edge” is horizontal or vertical, observers should (ideally) never respond with “left” or “right” for a horizontal fuzzy edge, or with “up” or “down” for a vertical fuzzy edge. Yet, they sometimes do; it happened in 0–31% of the cases. Nine observers show more than a 1% fraction of such slips, we mark them as “type B observers.”. Notice that it is perfectly possible that these type B observers are indeed unable to detect the transition between the two hues. But, although admittedly possible, we doubt it on the basis of our own experiences. Type B observers are perhaps to be considered “sloppy.”

For each observer, we determine the best linear order for the depth of the hues. We do this by way of a simple voting procedure. For each hue, we count how many times the hue was judged as closer. This yields a voting order.

Given the voting order, we test whether observers respond systematically on a given hue-pair independently of orientation. We find that observers vary widely in the coherence of their data, with one, evidently extreme, observer essentially responding randomly. The coherence is expressed in terms of the figure of merit, which is defined in analogy with Kendall's tau, on the basis of the number of actual judgments that were correctly postdicted on the basis of the fitted linear order. The figure of merit theoretically ranges from 0 to 1. We find values ranging from 0.03 to 0.83, the median is 0.61.

As a first step in grading the data, we defined the lower quartile range of the figure of merit as “type C observers,” and set them apart from the main overall analysis. Notice that it is perfectly possible that these type C observers have no particular association between depth order and hue differences at all. In the context of our study, they might perhaps be considered “weak” observers. The lower quartile value for the figure of merit is 0.383. We find that there are nine type C observers.

**Table 1.** At left, the distribution over the two clusters and the observers from the two locations. At right, the distribution under assumption that the location is irrelevant, using the overall mean for the distribution over the clusters.

	Trento	Leuven		Trento	Leuven
Cluster I	4	12	Cluster I	5 ± 0.2	11 ± 0.3
Cluster II	4	6	Cluster II	3 ± 0.2	7 ± 0.3

The significance limit for the figure of merit, as determined through simulation of a virtual “random observer,” is 0.27. We find that four of the nine “type C observers” have figures of merit that are not significantly different from 0. Moreover, four of the nine type C observers are also in the “type B” category. One of these observers does not respond significantly different from a simulated fully random observer. We take this as an indication that these nine observers should be excluded from the main overall analysis of the data, so we decided to treat them as a special group. Seven of these are from the Trento location (47% of the Trento observers). two from the Leuven location (10% of the Leuven observers). This is perhaps not too surprising, given the fact that the Trento group was composed of starting students working for credits, whereas the Leuven group was composed of postgraduate students, postdocs, and staff working in vision research.

Of the 35 observers there are 21 that are neither type B, nor type C. We refer to them as “type A observers”. Notice that “type B” and “type C” may mean that these observers have difficulty detecting the orientation of the division between the hues, and/or that they have no associations between depth order and hue differences. The terms are only used in the context of the method, in order to sort varieties of observers.

The second step is more complicated. An initial analysis of the individual observers suggested to us that they come in two categorically different groups. In order to investigate this, we performed a cluster analysis using the squared Euclidean distance function on the votes, and applying Mathematical default method. This indeed results in two clusters of roughly similar size (16 and 10). There is no marked relation between location (Trento or Leuven) and cluster membership (Table 1).

**Figure 6.**
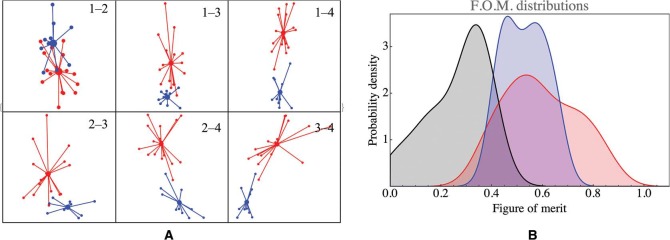
Left: The projections of all observers on a number of principal component planes. The observers in cluster I plotted in red, those in cluster II in blue. The centers of the clusters were defined as their barycentra. Notice that the first four principal components explain more than 80% of the variation in the voting orders, thus this figure yields a fair impression of the clusters. Right: Smooth histograms of the figure of merit (FOM) distribution over the type C observers, and the two clusters (black: type C observers; red: cluster I; blue: cluster II).

We find that the clusters are well separated (Figure 6 left) as seen from a variety of directions. Intercluster distances between point pairs are significantly larger than intracluster pairwise distances. Apparently, there exist two categorically distinct groups of observers. Either cluster is evidently distinct from the set of type C observers (Figure 6 right).

We discuss the data for the three groups (type C observers, cluster I, cluster II) separately. Notice that both the detection of the type C observers, as well as the clustering was done fully automatically, without any subjective intervention.

## 4 Analysis

### 4.1 Type C observers

This group involves nine participants. Four of these are also in the “type B” category. One observer cannot be differentiated from an observer responding randomly.

The color fuzzy edge is evidently not the only depth cue. An obvious candidate is position in the picture. One expects the lower side to have a bias to be seen as “nearer.” There is indeed evidence that this played a role. From the 2.376 responses, we counted

- 731 lower side
- 460 upper side
- 549 left side
- 636 right side

cases, whereas one expects 594 in each case. We expect observers to make a binary choice for each trial, that is either between left side/right side, or between lower side/upper side. Thus, the standard deviation from the binomial distribution is 17.2. Apparently, position in the picture is a rather powerful cue. The lower side is much more likely to be judged closer than the upper side, the difference is almost 16 standard deviations. Notice that there is also an overrepresentation of right over left of about 5 standard deviations.

Of course, we designed our method to be insensitive to these strong biases, which were to be expected.

The voting orders for this group of observers do not mutually correlate very well (pairwise Kendall tau's vary between −0.75 and +0.85), as is to be expected since their individual figures of merit are very low to begin with. The median value of 0.17 is not significantly different from zero (the standard deviation for random observers is 0.23). A conservative assessment is that this group of participants gives no reason for one to assume that color is a significant factor in the observed depth-orders at all.

### 4.2 Cluster I

This group involves 16 participants. Three of these observers are in the “type B” category.

We again check the distribution over sides. From the 4.224 responses, we counted

- 1.241 lower side
- 866 upper side
- 1.055 left side
- 1062 right side

cases, whereas one expects 1.056 with standard deviation 23.0 for each case. Apparently, position in the picture is a rather powerful cue. The lower side is much more likely to be judged closer than the upper side, the difference is more than 16 standard deviations. Right-left bias is not significant, the difference being less than a third of the standard deviation.

As expected, the orders derived for the participants in this cluster correlate well with each other. The range of the pairwise rank correlations (Kendall's tau) is −0.27 to 0.89. median 0.44. interquartile range from 0.27 to 0.61. The lowest value is evidently an outlier, being lower than the 5% quantile (which is −0.09).

**Figure 7.**
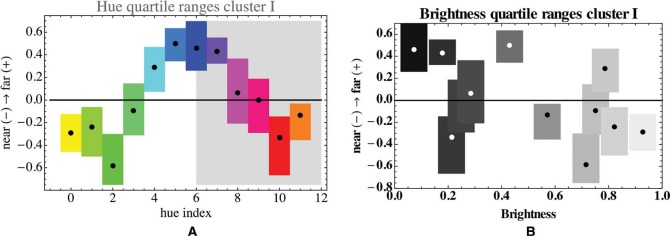
Left: The linear depth order for the pooled participants of cluster I, as a function of the hue index. The hues have also been indicated by their color. The boxes show the interquartile ranges, the dots the median value. Only the nonshaded region represents ecologically relevant hues. Right: Here, the same relation is shown for the brightness of the colors.

In Figure 7. we show the overall linear depth order for the cluster as a whole. The interquartile ranges are much smaller than the total range, thus the hue order indeed “resolves” the depth range to some extent. The brightness of the colors carries hardly any relation to the height in the picture.

The hue index range between 0 and 5 occurs in the averaged landscape photographs. In this range, height in the picture increases monotonically with the hue index (Kendall's tau 0.73; *p*-value 0.011). There appears to be no obvious relation between height and brightness (Kendall's tau −0.36; *p*-value 0. 038). Note that the rank correlation is negative in this case (see below).

### 4.3 Cluster II

This group involves 10 participants. Two of these observers are in the “type B” category.

We again check the distribution over sides. From the 2,640 responses, we counted

- 802 lower side
- 511 upper side
- 623 left side
- 704 right side

cases, whereas one expects 660 with standard deviation 18.2 for each case. Apparently, position in the picture is a rather powerful cue. The lower side is much more likely to be judged closer than the upper side, the difference is about 16 standard deviations. There is also an overrepresentation of right over left, of a little over 4 standard deviations.

As expected, the orders derived for the participants in this cluster correlate well with each other. The range of the pairwise rank correlations (Kendall's tau) is −0.03 to 0.88, median 0.53, interquartile range 0.38-0.64.

**Figure 8.**
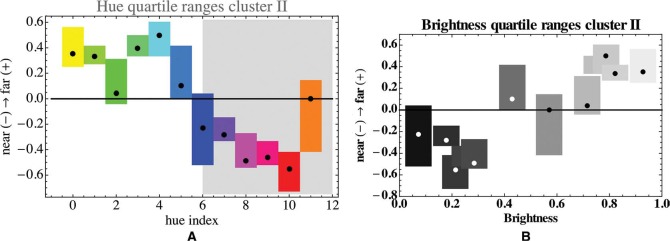
Left: The linear depth order for the pooled participants of cluster II, as a function of the hue index. The hues have also been indicated by their color. The boxes show the interquartile ranges, the dots the median value. Only the nonshaded region represents ecologically relevant hues. Right: The distribution of brightness. We used a linear luminosity function with relative weights *R: G: B* = 3: 6: 1 (Stokes, Anderson, Chandrasekar, & Motta, 1996), but this is not at all critical.

In Figure 8, we show the overall linear depth-order for cluster II as a whole. The interquartile ranges are much smaller than the total range, thus the hue order does “resolve” the depth range to some extent. The order in the range of hue indices 0–5 is indeterminate (Kendall's tau 0.07; *p*-value 0.42). For cluster II, the brightness increases monotonically with the height in the picture (Kendall's tau 0.64; *p*-value 0.002). Note that this contrasts with the direction of the correlation in Cluster I.

### 4.4 Overall analysis

We obtain three groups, a group of participants for which hue and depth appear unrelated (type C), and two groups (clusters I and II) for which there is a significant relation, although the latter two groups are mutually quite different.

**Figure 9.**
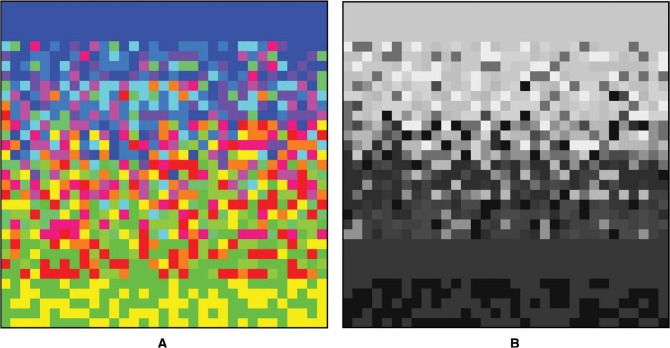
Left: This image was computed by randomly sampling from the empirical distribution defined by the hue results from cluster I. Right: This image was computed by randomly sampling from the empirical distribution defined by the brightness results from cluster II.

Cluster I reveals a hue-induced depth order that apparently reflects the “ecological order” of hues, whereas cluster II reflects the “ecological order” of brightnesses, as can be seen from the simulation result in Figure 9.

In the hue distribution of cluster I (Figure 9 left), the reds and magentas also occur, these do not occur in the analysis results of the landscape images. The reds occur mainly in the bottom half, the purples in the midrange. This is indeed to be expected on generic ecological grounds, since these colors are due to various stray foreground details, such as flowers. We have no statistics to corroborate this speculation.

**Figure 10.**
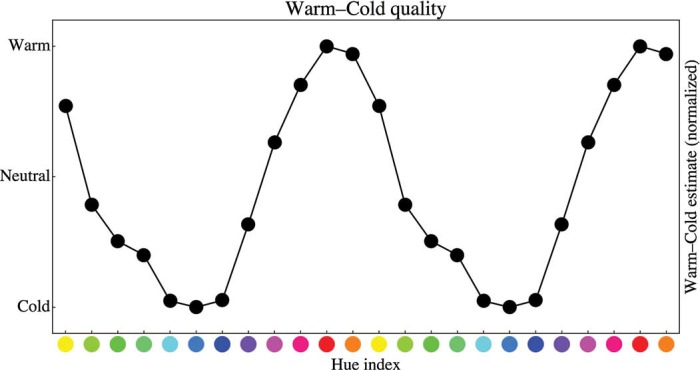
Judgment of color quality on a warm–cold scale. Warm is up, cold down. Red is the warmest color, blue the coldest. Green and magenta are neutral. Notice that we plotted two full hue-index cycles for the sake of visual clarity.

An alternative explanation for the hue dependence will immediately occur to those with some background in color science. It is generally understood in the art world that “warm colors come forward, cold colors recede” (Chevreul, 1854; Da Pos & Valenti, 2007; Guibal & Dresp, 2004; Gurney, 2010; Itten, 1961; Luckiesh, 1918; Payne, 1964; Sundet, 1978; Troscianko, Montagnon, Le Clerc, Malbert, & Chanteau, 1991; von Allesch, 1925). We determined the responses of 37 observers (a different group from the participants in the present study, all from the University of Trento) on a five-point “warm” to “cold” scale. Perhaps surprisingly, this yields very coherent data (Figure 10). Apparently, even a group of fully naïve observers is spontaneously able to make warm–cold distinctions.

**Figure 11.**
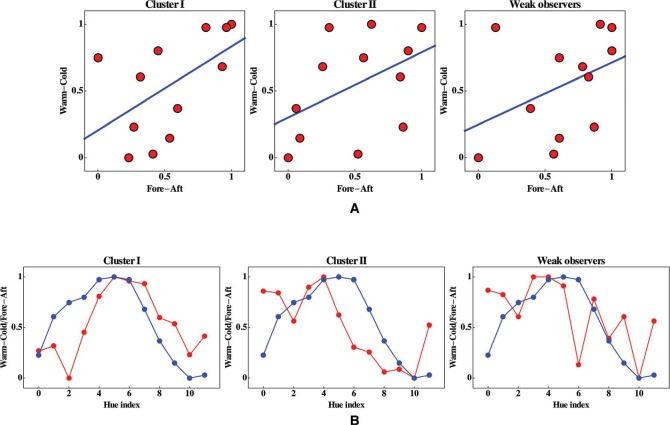
Top: Scatterplots of the median fore-aft against the warm–cool values for the three groups. All values were normalized to the [0,1] range, in order to aid in the visualization. The blue lines are best linear fits, their slopes are 0.63 (cluster I), 0.49 (cluster II), and 0.46 (weak observers). The Kendall's tau rank correlations are 0. 41 (cluster I), 0.42 (cluster II), and 0.39 (weak observers). Spearman's Rho's are 0.68 (cluster I), 0.56 (cluster II), and 0.55 (weak observers). Bottom: Here, the median fore-aft (red) and warm–cool (blue) values have been plotted both, as a function of the hue index. One believes to spot “phase shifts” that are quite different for the various clusters.

In Figure 11 top, we present scatter plots of the range-normalized fore-aft against the warm–cool values for all clusters. None of the rank correlations is impressive. Given that the Kendall's tau rank correlations between weak observers range from −0.75 to 0.85, one concludes that the warm–cold dimension is irrelevant for this group. For both clusters I and II, more than half of the correlations between observers is higher than that between the warm–cold values and the median fore-aft values. The highest interobserver correlations are more than 0.8, whereas the correlation with the warm–cold dimension is only 0.41 (cluster I) or 0.42 (cluster II). The latter values are slightly larger than the 95% quantile of the rank correlation between two random observers, which is 0.364. Thus, there exists only a marginal correlation.

A combined plot of the median fore-aft values and the warm–cool values against the hue index (Figure 11 bottom) suggests that the rank correlations may not reveal the full story, since a well-chosen “phase shift” would very likely increase them significantly. We return to this issue in the general conclusions section.

The so-called “chromostereopsis” (Kishto, 1966), mentioned above, is another factor to consider. It is due to the influence of the chromatic aberrations of the eye. Three core cluster I observers did the experiment both monocularly and binocularly, with no significant difference in the result, which already suffices to rule the effect out as a relevant causal factor. Moreover, chromostereopsis fails to explain the fact that we find distinct clusters of observers, since all observers necessarily agree in the chromatic aberration of their eyes^1^. Furthermore, we find no significant differences between the vertical and horizontal presentations, so disparity does not play a role.^2^ In the pattern of cluster II, red is nearest, yellow and cyan most remote, blue in the middle, which is fully at odds with the chromatic aberration predictions. Thus, we are convinced that chromostereopsis fails to account for our findings.

## 5 Conclusions

Our results clearly indicate that hue differences evoke impressions of depth-order in the majority of observers. This happens independently of factors such as orientation, or location in the image. As an objective empirical fact, one might take it as the main result of the study.

A striking effect, present in all participants, is that the lower side of an image is far more likely to be judged closer than the upper side is. This is perhaps not all that surprising, but it is certainly a striking feature of our data. It is unlikely to have to do with color, because informal observations reveal the same effect in monochrome photographs, or even line drawings. Although generally acknowledged, and often mentioned in drawing or painting instructions, we have not seen it documented from extensive psychophysical data. More surprising is that the right side is more likely to be judged closer than the left side in all cases, except for cluster I. Although less marked than the top–bottom asymmetry, this is also a remarkable bias.

The strong top–bottom bias is very likely due to a major ecological asymmetry, related to the basic fact that we tend to view the scene in front of us while standing upright—at least most of the time. It is evidently a template structure deployed in the psychogenesis of visual awareness.

The left–right bias, does not easily yield to such an argument. It might well be a response bias. Otherwise, one expects that it might be culturally determined, but we really can't say. Only cross-cultural studies might prove the validity of such expectation/assumption.

The standard account in the visual arts is that warm comes forward, whereas cool recedes. This is used in the articulation of pictorial relief by spatially selective “warming” or “cooling” the color. We do not clearly see that in the results, there exists only a minor correlation. On the basis of this finding, we did not pursue this hypothesis.

Of course, it is not evident why the warm–cold dimension should be interpreted to at least partially “explain” the fore-aft dimension, instead of the other way around. We notice merely some correlation.

Although the correlations between the fore-aft and warm–cold dimensions are indeed minor. “eye-balling” the combined plots shown in Figure 11 bottom suggests that there might be a relation different from a mere monotonic dependence. It appears that there exists a “phase shift” between these relations that accounts for much of the low correlation. This shift is in different directions for clusters I and II.

The shifts are quite large, amounting to the difference between yellow and green. This seems a potentially rewarding topic for future research. The “warm–cold” dimension is likely to be another template whose evolutionary origin deserves to be studied.

**Figure 12.**
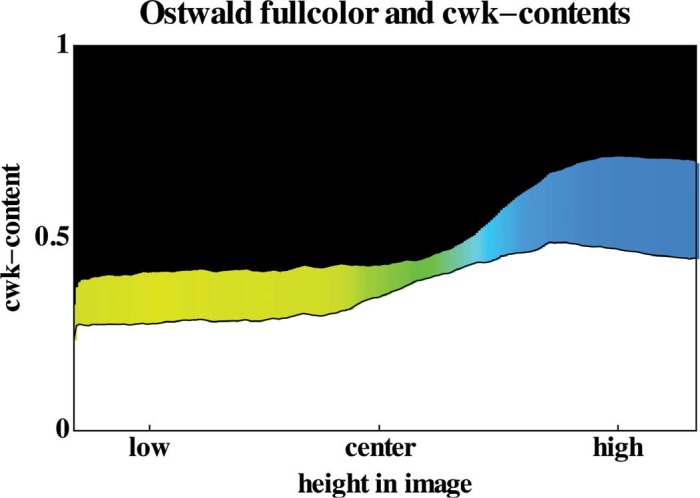
The Ostwald color, white, and blue contents, as well as the hue of the full color, as a function of height in the image (see Appendix B on the Ostwald color description). These statistics are based on the database of 144 open landscape photographs from Torralba's SUN Database (Xiao et al. 2010).

It is tempting to speculate that the hue dependence reported in this study might also be related to ecological factors. Of course, there is not really a way to show this conclusively, it can at best be forged into a reasonable proposition. Although we do not have the extensive databases one would prefer, it is at least possible to predict the hue order from theoretical ecological physics. Moreover, there exist plenty of photographs on the Internet that allow one to collect some statistics. The distribution of white, black, and color content, including hue, for the set of 144 open landscape photographs (Xiao et al., 2010), is shown in Figure 12. It is evident that both the yellow–blue and the black–white gradients correlate well with the low-high position in the image.

We find that the statistics on sets of random landscape photographs yield very robust results (Figure 12), which correlate well with the expectations from theoretical ecological optics. When confronting such data with our data, we find that there exist two groups of observers, roughly of equal size, such that one (our cluster I) correlates with the predicted hue dependence, and the other (our cluster II) with the predicted brightness dependence.

So much for the facts, what to make of them? We venture that many human observers have either a generic hue, or a generic brightness—or perhaps both—template that shows up in the results of their psychogenesis of visual awareness. Such a template might act as a “gist” that could serve to start up an open landscape oriented “analysis by synthesis” cycle (Neisser, 1976). Confronted with the “abstract paintings” in our experiment, this might well lead to the observed effects. Such a template might conceivably be a remnant from the period that early prehumans roamed the African savannah (String, 2011).

In order to investigate such a possibility, it would be of interest to study the influence of field size, whether observers have both a hue and a brightness template depending upon the situation, and so forth. It is perhaps remarkable that we detect significant trends even in the case of only moderately large pictures. One would expect such trends—if any—primarily in large field displays. It is equally remarkable that the template works in other than the “standard” orientation. Moreover, it seems a priori likely to us that the two modes—hue and brightness—are part of the make-up of virtually any observer, and that the dichotomy here is at least partly induced by the particular setting. This suggests a number of further studies, involving a much more extensive sample from the population than could be used here.

We suggest the existence of a generic “landscape template” in human vision. Of course, this cannot be more than a suggestion. This should not detract from the main finding of the study though: most human observers associate depth order with hue differences or brightness gradient in a remarkably systematic manner.

## Figures and Tables

**Figure 13.**
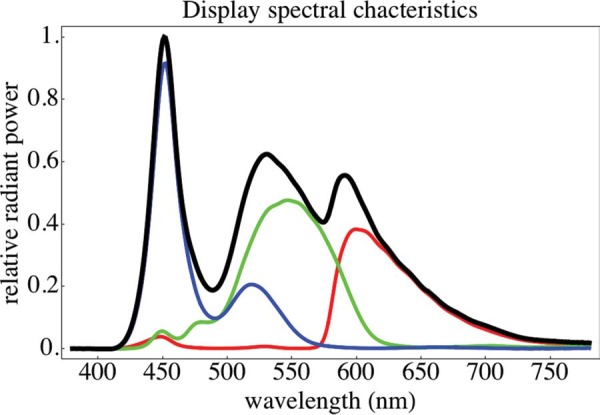
The radiant power spectra (normalized to the maximum of the combined-white-spectrum) of the red, green, and blue color channels of the display. Spectral resolution is 1 nm, random error of relative radiant power within about ±1%. These spectra are relevant if one desires to explain our results as due to chromatic aberrations of the eye optics. As explained in the main text, we have compelling reasons not to accept such an explanation. The color channels are drawn in red, green, and blue, the combined white is indicated with the black curve.

**Figure 14.**
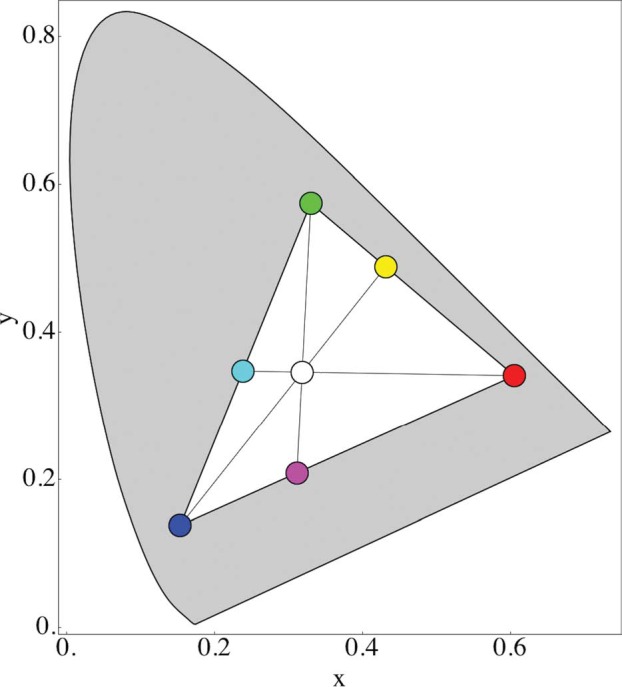
Here, we have plotted the primary colors (red, green, and blue color channels) as well as the secondary colors (cyan = green + blue, magenta = blue + red, and yellow = red + green) in the conventional 1931 CYE xy-diagram. This might be important for those who seek the explanation for our results in the precise colorimetric data.

**Figure 15.**
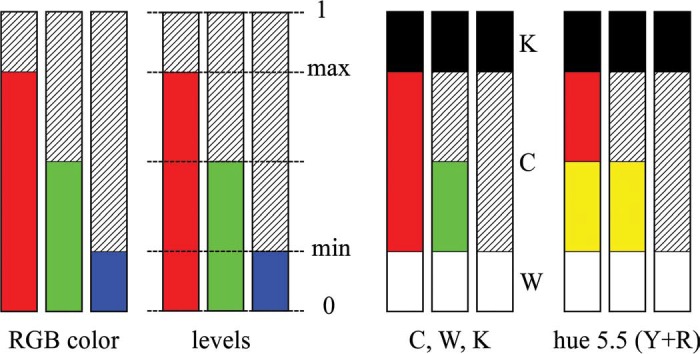
An example of the Ostwald analysis of RGB colors. At left, an RGB color, presented as a bar diagram. The second subfigure shows the relevant levels, the third the color, white, and black contents. In the fourth subfigure, the full color has been split into the primary (here red), and the associated secondary component (here yellow). The color is orange, an equal mixture of red and yellow, with 20% black and 20% white. The Ostwald designation is {5.5, {0.6, 0.2, 0.2}}.

## Footnotes

1 The chromatic aberration is due to the dispersion of water, which is the effective optical medium for the cornea, lens, and vitreous body.

2 We tested the blue/cyan against the red/yellow pairs, the two spectral limits. This involves 128 cases, and should show up possible chromatic aberration effects. The Wilson score intervals (Wilson, 1927) for the vertical and horizontal separations overlap by 56% at the 5% level. Thus no chromatic aberration effect is detectable.

## Appendix A: Spectrophotometric calibration of the display

The display was calibrated in the following way:

First, we linearized the display using the SuperCal application of BergDesign (2014). setting the gamma to 1.0. the white point to D65. We believe this method to be probably more precise than a straight radiometric one, which would depend upon the accuracy of the radiometry at low radiances, Anyway, the results should be the same. Leaving these settings intact—they are not used any more—we did a full spectroradiometric calibration. We only mention the relevant data here.

For the radiometric measurements, we used a Minolta CS-1000 (Minolta. 2015) in a fully dark environment (matte black painted walls and so forth). taking various measures to avoid artifacts. We limited spectroradiometry to the 380–780 nm spectral region. Unfortunately, we had to rely on the factory calibration since we had no access to a secondary standard, such as the usual incandescent tungsten ribbon pyrometric lamp (Rutgers. 1972). Spectral resolution was set to 1 nm, which is sufficient to capture most structure in the LCD-screen radiant power spectrum.

We calibrated only the center part of the screen—about 5% of the stimulus width—but we know from spatially selective luminance measurements that the uniformity is much better than is required for the experiment. The edges of the screen remain unused.

A realistic assessment of the remaining uncertainties is as follows:

The factory guarantees an accuracy of ±4% in luminance and ±0.001 in the chromaticity coordinates. We have not checked the polarization error (which might well be important for LCD screens) but the factory guarantees it for better than ±5%. In view of the design, this seems trustworthy, since the path of the measuring beam involves no reflections due to a hole in the reflex-viewer-mirror. However, we are not sure whether the objective lens was included in the factory calibration.

All in all, we feel that the chromaticities might be trusted to up to two decimal numbers and the luminances to within ±10%. These accuracies exceed those needed for the correct interpretation of our results by a very wide margin.

We performed radiometric measurements for black, white, the primary colors, and the secondary colors. This twofold overdetermination allows us to check on linearity and possible nonlinear crosstalk between channels.

The major findings are:

The dynamic range is between 400 (for blue) and 800 (for green). This is fairly typical (Soneira. 2015) for these LCD displays. In the actual experiment, the effective dynamic range was not limited by the stray light in the room.

The linearity turns out to be so good that deviations cannot really be captured at the level of accuracy reported here. The radiant power spectra of the primary colors (red, green, and blue channels) are shown in Figure 13.

The corresponding chromaticity coordinates using the CIE 1931 definition and the luminances (in candela per meter square) are given in Table 2.

**Table 2.** The chromaticity coordinates *x, y* and the luminances *Y* of the red, green, and blue color channels. We would trust at least two digits of the chromaticity coordinates and the luminances to within ±10%. These data might be of interest for those who are convinced that more luminous implies closer. We do not harbor such conviction and we feel certain that our data do not imply anything of that kind.

	*x*	*y*	*Y*
Red	0.6049	0.3416	94.3
Green	0.3295	0.5750	272.7
Blue	0.1531	0.1382	71.1

Notice that the luminances are in the ratio R: G: B = 0.215: 0.622: 0.163. thus very close to the usual rules of thumb. Of course, that can hardly be otherwise, since the primary colors combine to white. Relative luminances can be estimated quite accurately using such a rule of thumb without any calibration at all.

The overall luminance level is indeed a relevant parameter. We have not repeated the experiment at a range of luminance levels. If the ecological interpretation makes sense, one expects only the highest luminance levels—daylight levels say—to be relevant. With the LCD display we are in a fairly low, though most likely relevant range. We are out of the photon shot noise dominated regime and well in the lower Weber range. Thus, it is not likely that variations by a factor of two or so would make a difference.

In Figure 14. we have plotted the primary and secondary colors in the 1931 CIE chromaticity diagram. Notice that we used six additional colors in the experiment that have not been plotted for the sake of clarity. Although these are indeed *between* the indicated colors, they do not bisect those. Remember that the CIE diagram is a projective transformation of the CIE XYZ color space. This conventional representation is much less intuitively useful than that used in the main text, we give it for completeness.

Although we believe that the calibration data reported here are not critical for the interpretation of our data, they might be of some use to those who prefer to stick to a chromostereopsis interpretation, which is why we present them. We consider such an interpretation to be extremely unlikely on the basis of our data—as argued in the main text—but we cannot definitively exclude it. However, we are quite certain that the converse could not be made to stick either. Anyway, with the spectroradiometric data in place anyone who feels like it is in a position to attempt to put us in the wrong.

## Appendix B: Ostwald color coordinates

Since not all readers may be familiar with the Ostwald color description (Koenderink. 2012; Ostwald. 1917). here is a summary account. We focus on RGB colors, for which the Ostwald system is particularly suited. (Ostwald himself focused on the spectrum, in his time there were no RGB-devices in common use.) “RGB colors” are specified by three numbers which we take to be in the range [0,1]. These specify the amounts of red, green, and blue in an image.

Consider a color *C = {r,g,b}* (example in Figure 15). Define the minimum *w* = min[*{r,g,b}*]. and the maximum *p* = max[*{r,g,b}*]. Then, define the white content as *w*, the black content as *k* = 1−*p*. and the color content as *c* = 1 − *w − k*. Thus, you have *c* + *w* + *k* = 1. The white part of the color is {*w,w,w*}. when we subtract it from {*r,g,b*}, we obtain a triple of numbers {*u,v,w*} = {*r,g,b*} - {*w,w,w*} (say) of which at least one is necessarily zero. Dividing by the color content yields a triple {*k,l,m*} = {*u,v,w*}/*c* of which one component is zero, one equal to unity, and the remaining one in the range [0.1]. Such a color might be denoted “full color.” since it contains neither white, nor black. The original color can be represented as *c F + wW*. where *F* is the full color {*k,l,m*}. and *W* = {*1,1,1*} is white. Notice that the black takes care of itself, since the highest coordinate value will be 1 − *k*.

For aesthetic reasons, one might prefer to set

$$\begin{array}{l} C=cF+wW+kK,\\ \;W=\{1,1,1\},\\ \;K=\{0,0,0\}, \end{array}$$

whereas

c+w+k=1,

where *F* is a full color. This is actually useful in some contexts, e.g., when one generates colors with inks on white paper, instead of with lights on darkness.

There are two special types of full color, namely the “primary colors” *R* = {1,0,0}, *G* = {0,1,0}, and *B* = {0,0,1}, and the “secondary colors” *C* = {0,1,1}, *M* = {1,0,1}, and *Y* = {1,1,0}. Any full color can be written as the unique combination (1 - μ) *P + μS.* where *P* is a primary color. *S* an associated secondary color, and μ a number in the range [0.1]. (For instance, orange is (1 - μ)*R* + μ*Y.* for μ = 0.5, that is {1, ½ 0}.) Here, “associated” means that the primary and the secondary colors share one zero coordinate. Thus. *R* is associated with *M* and Y. *G* with *Y* and C, and *B* with *C* and M. Purely formally, this implies that the “cardinal colors” can be arranged in the cyclical order *…YGCBMR….* the so-called color circle. It is indeed a topological circle, though perhaps better regarded as a hexagonal outline. One conveniently defines a “hue index” starting with 0 for *Y*, and going over *G*. Thus. *R* gets hue index 5. whereas *Y* would get 6 (the next step after R). but because of the periodicity we have 6 = 0.

Thus, a full Ostwald specification of an RGB-color involves the hue index, and the color, white, and black contents (c, *w, k* under the constraint *c + w + k* = 1).

Then, the Ostwald coordinates of an RGB color are {*h*, {*c,w,k*}}, where *h* denotes the hue index, *cwk* the color, white, and black content, under the constraint *c + w + k* = 1. The result is simple to calculate, and has immediate intuitive meaning. With a little experience, you can actually guesstimate hue index, color, white, and black content pretty well by eye.

Given this setup, it is easy to find “how much” of a given hue range is contained in an image. One finds all pixels with hue index in the range, and adds their color content to the total. Divided by the total number of pixels in the image, one obtains a number that specifies “how much.” In a similar manner, one measures the total amounts of black, or white.
